# Phenotypic and genotypic comparison of ESBL production by Vaginal *Escherichia coli* isolates from pregnant and non-pregnant women

**DOI:** 10.1186/1476-0711-12-7

**Published:** 2013-04-25

**Authors:** Sareaa MG Al-Mayahie

**Affiliations:** 1 Medical Microbiology, Department of Biology, College of Science, University of Wasit, Al-Kut City, Wasit Province, Iraq; 2 Iraq, Ministry of Higher Education and Scientific Research, University of Wasit, College of Science, Department of Biology, Al-Kut City, Wasit Province, Iraq

**Keywords:** ESBL, Vaginal *E. coli*, Comparison

## Abstract

**Background:**

Vaginal *Escherichia coli* is a reservoir along the fecal-vaginal-urinary/neonatal course of transmission in extraintestinal *E. coli* infections. They also causes genital tract infections especially vaginitis, so that detection of their antibiotic resistance is an important approach to control these infections. One important mechanism of resistance is ESBL production by Enterobacteriaceae especially *Klebsiella* spp. and *Escherichia coli*, which is now a worldwide problem that limits therapeutic options.

**Methods:**

Sixty one vaginal *E. coli* isolates from pregnant and non-pregnant women, were detected phenotypically and genotypically for ESBL production.

**Results:**

Most of pregnant and non-pregnant women's isolates, were resistant to cefotaxime (100% vs. 81.5%, respectively) and more than half of them to ceftazidime (56.5% vs. 71.0%, respectively). One hundred percent each, 52.1% vs. 68.4%, and 73.9% vs. 60.5%%, were ESBL producers by screening, confirmatory, and PCR tests, respectively. Pregnant women's isolates had: CTX-M- (69.5%), SHV- and OXA-type (each 4.3%) ESBLs. Only one isolate (4.3%) had two types of ESBLs. All 16 CTX-M-positive (100%) isolates had CTX-M-1. Non-pregnant women's isolates were predominated by SHV and CTX-M -type (44.7% vs. 39.4%, respectively), followed by OXA- (15.7%), and TEM-type (2.6%). Of these isolates, 42.1% had two types of ESBL genes. All 15 CTX-M-positive (100%) isolates had CTX-M-1. Pregnant and non-pregnant women's isolates differed significantly (P≤ 0.05) regarding the expression of SHV- (4.3% vs. 44.7%, respectively) and CTX-M-type (69.5% vs. 39.4%, respectively) ESBLs. In both, CTX-M-1 was the predominant CTX-M group **(**each 100%). All of the isolates were susceptible to imipenem and meropenem, while the highest rate of resistance was against β-lactams. Multidrug resistance was noted in 56.2% of ESBL-producing isolates.

**Conclusions:**

Ggenital tracts of pregnant and non-pregnant women represent different environments for propagation of ESBL-producing *E. coli*. In Iraq, nationwide study is required to make a precise estimation of this widespread of ESBL-producing bacteria.

## Introduction

*Escherichia coli* is one of the common organisms in the vaginal microflora of pregnant as well as non-pregnant women and it may also cause symptomatic infections [1] such as vaginitis or tubo-ovarian abscess and is associated with life threatening neonatal sepsis and meningitis [2,3].

The emergence of resistance to antimicrobial agents continues to evolve substantially, influencing the evaluation and treatment of infections in nosocomial and health care–associated settings and in the community. Bacteria use several strategies to avoid the effects of antimicrobial agents, and have evolved highly efficient means for clonal spread and for the dissemination of resistance traits [4]. Extended-spectrum β-lactamases (ESBLs) are capable of hydrolyzing broad spectrum cephalosporins and monobactams. In addition, ESBL producing organisms exhibit co-resistance to many other classes of antibiotics resulting in limitation of therapeutic options. The resistant organisms are now a worldwide problem [5-7] and their incidence is being continuously increasing with limited treatment alternatives. It becomes necessary to know the prevalence of these organisms and to formulate treatment policy [5]. Vaginal *E. coli* represents a real threat specially to neonates, however, few information is available regarding their antibiotic resistance, therefore; this study was carried out to detect and compare, phenotypically and genotypically, ESBL production by *E. coli* isolates from pregnant and non-pregnant women.

## Material and methods

### Patients and bacterial isolates

This study included sixty one isolates of *E. coli* recovered as significant growth from high vaginal swabs collected from pregnant and non-pregnant women (aged 18–45 years) clinically diagnosed as having symptomatic genital tract infection. The symptoms were characterized by purulent vaginal discharge and were recurrent despite treatment, as it was made clear by the Gynecologists. The exact cause of infection was not further investigated in this study. These isolates were collected over a 2-year period from May 2008 to June 2010 at Obstetrics and Gynecology Clinics in Al-Kut City/Wasit Province/Iraq, and were identified by conventional biochemical tests [8,9]. Performance of this research was approved by the Wasit Health Administration/ Wasit Province / Iraq.

### Susceptibility testing

Disk-diffusion tests were carried out with antibiotic-containing disks (Bioanalyse) on Mueller-Hinton agar plate (Himedia). The results were expressed as susceptible or resistant according to the criteria recommended by the Clinical Laboratory Standards Institute (CLSI) [10]. The following antimicrobial agents were tested: gentamicin (CN: 10 μg), kanamicin (K: 30 μg), ciprofloxacin (CIP: 5 μg), norfloxacin (NOR: 10 μg), trimethoprim-sulfamethoxazole (SXT: 1.25/23.75 μg), imipenem (IMP: 10 μg), meropenem (MEM: 10 μg), amoxicillin-clavulanic acid (AMC: 20/10 μg), cefotaxime (CTX: 30 μg), ceftazidime (CAZ: 30 μg ), ceftriaxone (CRO: 30 μg ), and aztreonam (ATM: 30 μg).

### Phenotypic screening for ESBL

Screening of reduced susceptibility to third generation cephalosporins was carried out using Cefotaxime (CTX) and ceftazidime (CAZ) discs and double-disk synergy (DDS) method was used to confirm the presence of ESBLs as recommended by the Clinical and Laboratory Standards Institute [10].

### PCR amplification for detection of β-lactamase genes

All isolates were screened for the resistance genes TEM, SHV, CTX-M, and OXA by a multiplex PCR assay using universal primers (Table 1) [11-13]. Each isolate was subcultured on trypticase soy agar plates for 24 h at 37°C. From the agar plate, 5 colonies were picked and suspended in 100 μl sterile distilled water. Bacterial suspensions were run for 10 min at 94°C [14] in a DNA thermocycler (MultiGene, Labnet International, Inc., USA) and cell debris were removed by centrifugation (12,000 rpm for 1 min). PCR amplification reactions were performed in a volume of 50 μl containing 25 μl of KapaTaq 2x Ready Mix (KAPA Biosystems, USA), 25 pmol concentrations of each primer, and 5 μl of DNA template. The cycling parameters were as follows: an initial denaturation at 94°C for 5 min; followed by 35 cycles of 94°C for 30 s, 45°C for 1 min, and 72°C for 1 min; and with a final extension at 72°C for 10 min. The amplified PCR products were subjected to electrophoresis at a 2% agarose gel in 0.5X TBE buffer.

**Table 1:** Nucleotide sequences of PCR primers used to amplify four ESBL genes

**Gene**		**Primer sequence (5'-3')**	**Amplicon size (bp)**	**Reference (s)**
*bla*_TEM_	F	AAACGCTGGTGAAAGTA	822	[11,12]
	R	AGCGATCTGTCTAT		
*bla*_SHV_	F	ATGCGTTATATTCGCCTGTG	753	[11,12]
	R	TGCTTTGTTATTCGGGCCAA		
*bla*_CTX-M_	F	CGCTTTGCGATGTGCAG	550	[11,12]
	R	ACCGCGATATCGTTGGT		
*bla*_OXA_	F	ATATCTCTACTGTTGCATCTCC	619	[13]
	R	AAACCCTTCAAACCATCC		

### Multiplex PCR for CTX-M phylogrouping

CTX-M-positive isolates were further analyzed for CTX-M phylogroups (CTX-M-1; -2; -8; -9 and −25/26) by multiplex PCR [15]. Primer pairs and predicted amplicon sizes were summarized in Table 2. Amplification conditions were: initial denaturation at 94°C for 5 min; 30 cycles of 94°C for 25 s; 52°C for 40 s and 72°C for 50 s; and a final extension at 72°C for 6 min.

**Table 2:** **Nucleotide sequences of PCR primers used to amplify *bla***_**CTX-M **_**alleles**

**Gene**	**Primer sequence (5'-3')**		**Amplicon size (bp)**	**Reference**
*bla*_CTX-M-1_	F	AAA AAT CAC TGC GCC AGT TC	415	
	R	AGC TTA TTC ATC GCC ACG TT		
*bla*_CTX-M-2_	F	CGA CGC TAC CCC TGC TAT T	552	
	R	CCA GCG TCA GAT TTT TCA GG		
*bla*_CTX-M-8_	F	TCG CGT TAA GCG GAT GAT GC	666	
	R	AAC CCA CGA TGT GGG TAG C		[15]
*bla*_CTX-M-9_	F	CAA AGA GAG TGC AAC GGA TG	205	
	R	ATT GGA AAG CGT TCA TCA CC		
*bla*_CTX-M_-_25/-26_	F	GCA CGA TGA CAT TCG GG	327	
	R	AAC CCA CGA TGT GGG TAG C		

### Statistical analysis

The Chi Square test was used for statistical comparison of groups; values < 0.05 were regarded as significant [16].

## Results

All of the vaginal *E. coli* isolates included in this study were susceptible to imipenem and meropenem, whereas all of them were resistant to amoxicillin-clavulanic acid. For both pregnant and non-pregnant women's isolates the highest rate of resistance was against β-lactams (Table 3), while resistance to other antibiotic's classes was moderate. Multidrug resistance was noted among 45.0% of all the isolates (36.8% and 50.0% of pregnant and non-pregnant women's isolates, respectively) and 56.2% of ESBL-producing isolates (50.0% and 61.1% of pregnant and non-pregnant women's isolates, respectively).

**Table 3:** **Resistance pattern of vaginal *E. coli *isolates to antibacterial agents**

Antibacterial agent^*^	**% of Resistant isolates**					
	**Pregnant women's isolates**		**Non-pregnant women's isolates**		**All isolates**	
	**Total (n = 19)**	**ESBL**^**+ **^**(n = 14)**	**Total (n = 32)**	**ESBL**^**+ **^**(n = 18)**	**Total (n = 51)**	**ESBL**^**+ **^**(n = 32)**
Gentamicin	36.8	50.0	18.75	27.7	25.4	37.5
Kanamicin	26.3	35.7	43.7	50.0	37.2	43.7
Ciprofloxacin	31.5	42.8	28.1	38.8	29.4	40.6
Norfloxacin	26.3	35.7	21.8	27.7	(21.5	31.2
Trimethoprim-sulfamethoxazole	31.5	42.8	53.1	72.2	45.0	59.3
Meropenem	0	0	0	0	0	0
Imipenem	0	0	0	0	0	0
Amoxicillin-clavulanic acid	100	100	100	100	100	100
Cefotaxime	100	100	78.1	91.3	86.2	93.7
Ceftazidime	57.8	76.4	71.8	78.2	66.6	81.2
Ceftriaxone	42.1	57.1	46.8	61.1	45.0	59.3
Aztreonam	42.1	57.1	46.8	61.1	45.0	59.3
**% Multidrug resistance**	**36.8**	**50.0**	**50.0**	**61.1**	**45.0**	**56.2**

* For all the tested antibiotics (tested later in the study), the total number of isolates was 51 (10 isolates were lost during storage as a result of electricity instability), except for cefotaxime and ceftazidime (tested early during the study) the number of isolates was 61.

ESBL production by these isolates also was detected and compared by phenotypic and genotypic procedures (Table 4).

**Table 4:** **Numbers (percentages) of ESBL producing vaginal *E. coli *isolates from pregnant and non-pregnant women**

**Characteristics**		**No. (%) of positive *E. coli *isolates**		
		**Pregnant women's isolates (n = 23)**	**Non-pregnant women's isolates (n = 38)**	**Total (n = 61)**
Resistance to:	CTX	23 (100): R:19; I:4	31 (81.5): R = 27; I = 4	54 (88.5)
	CAZ	13 (56.5): R: 11; I: 2	27 (71.0): R = 26; I = 1	40 (65.5)
Phenotypic detection of ESBLs:	Screening test	23 (100)	38 (100)	61 (100)
	Confirmatory test (DDST)	12 (52.1)	26 (68.4)	38 (62.2)
Genotypic detection of ESBLs *(bla* genotype):	CTX-M	16 (69.5)	15 (39.4%)	31 (50.8)
	SHV	1 (4.3)	17 (44.7)	18 (29.5)
	TEM	0	1 (2.6)	1 (1.6)
	OXA	1 (4.3)	6 (15.7)	7 (11.4)

ESBLs: extended spectrum β-lactamases; CTX: cefotaxime; CAZ: ceftazidime; R: resistant; I: intermediate resistant; DDST: double disc synergy test.

All pregnant women's isolates (100%) were resistant to CTX and more than half of them (56.5%) were resistant to CAZ. All isolates (100%) were suspected producers of ESBLs and 52.1% were confirmed phenotypically to be ESBL producers. ESBL genotypes were detected in 73.9% (17/23) of these isolates. CTX-M-type ESBL was the most common (69.5%) followed by SHV- and OXA-types (each 4.3%). None of these isolates had TEM-type ESBL. Only one isolate (4.3%) had two types of ESBLs (CTX-M- and SHV-type) and it was resistant to both CTX and CAZ. All CTX-M-positive (100%) isolates had only CTX-M-1.

For non-pregnant women's isolates, phenotypically, 81.5% and 71.0% were resistant to CTX and CAZ, respectively. All of them (100%) were suspected producers of ESBLs, whereas 26 (68.4%) were ESBL producers by confirmatory test (Table 3). ESBL genotypes were detected in 60.5% of these isolates. All four ESBL genotypes were found with predominance of SHV- and CTX-M-type (44.7% vs. 39.4, respectively), followed by OXA- (15.7%), and TEM-type (2.6%). Of these isolates, 16/38 (42.1%) had two types of ESBL genes: ten (10/16: 62.5%) were SHV- and CTX-M-positive; five (5/16: 31.2%) were OXA- and CTX-M-positive and one (1/16: 6.2%) was OXA- and SHV-type positive. All isolates with two types of ESBLs were resistant to both CTX and CAZ except one isolate with genotype of SHV- and OXA-type, which had intermediate resistance to CTX and was sensitive to CAZ. CTX-M-1 was expressed by all CTX-M-positive isolates.

**Figure 1:**
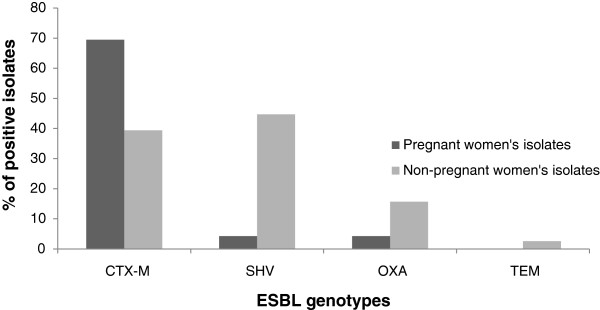
**Comparison of ESBL genotypes' distribution among vaginal *E. coli *isolates from pregnant and non-pregnant women.**

Pregnant and non-pregnant women's isolates differed significantly (P≤ 0.05) regarding the expression of SHV- (4.3% vs. 44.7%, respectively) and CTX-M-type (69.5% vs. 39.4%, respectively) ESBLs (Figure 1). One (2.6%) of non-pregnant women's isolates had TEM-type ESBL, whereas none of the pregnant women's isolates had this ESBL genotype.

## Discussion

This study was designed to detect and compare ESBLs' distribution among vaginal *E. coli* isolates from pregnant and non-pregnant women. Most of this study-included isolates were resistant to CTX and more than half of them were resistant to CAZ. All of CAZ resistant isolates were also resistant to CTX. The members of Enterobacteriaceae possess many mechanisms of resistance to β-lactam antibiotics, however, β-lactamases are the most common and clinically significant mechanism of resistance to β-lactam antibiotics among this bacterial group [7]. Four types of ESBLs were detected in this study, namely: TEM-, SHV-, CTX-M-, and OXA-type. Of this study-included isolates, 65.5% (40/61) were ESBL producers by PCR technique. In Iraq, previous researchers [17,18] reported ESBL production by 100% of enteropathogenic *E. coli* (EPEC) isolates from infants, most of them were suffering from watery diarrhea for more than 10 days, for that they were hospitalized. The important reason for this difference with these researchers was that they dealt with hospitalized patients and it is well known that the main source of ESBL producers was nosocomial and also even recent hospital contact may lead to colonization with these ESBL producers [4,19]. In addition, the prevalence of ESBLs differs among patient groups and clinical and geographic settings [20]. This high ESBL frequency reported in this study and in previous studies [17,18] is due mainly to the lack of control over prescription and sales of antibiotics here in Iraq, together with a lack of attention to laboratory screening of ESBL production by clinical isolates. Only at the level of academic research at the Universities, this subject is recently under study. In comparison with neighboring countries, this high percent of ESBL production noted in this study, is in agreement with what is found in Iran where 56% [21] of *E. coli* isolates were ESBL producers, and in Turkey where 74.6% [22] of *E. coli* isolates from hospitalized patients, and 36.7% of isolates from outpatients [23] were ESBL producers. While it is higher than that noted in Arab Gulf countries, such as Saudi Arabia [24] and Kuwait [25] (10% and 26% of hospital isolates, respectively, and 4% and 12% of community isolates, respectively). These geographical differences in the rates of ESBL production from country to country and even within countries from hospital-to-hospital, were reviewed by others [19,20].

The most common ESBL type found in this study was CTX-M, followed by SHV and OXA types, while TEM-type was rare. This result is consistent with the present situation in most parts of the world, where CTX-M-type have replaced TEM and SHV types [26] and became the predominant ESBL among Enterobacteriaceae in most parts of the world including the Middle East area, where they are prevalent not only in nosocomial environment, but also in the community setting [19,20,27]**]**. The high distribution of CTX-M-type ESBLs (50.8%) and CTX-M-1 (50.8%) among this study's isolates explains the high rate of resistance to CTX (88.5%) in comparison to CAZ (65.5%), since CTX-M β-lactamases, in contrast to most TEM and SHV ESBLs, preferentially hydrolyze cefotaxime over ceftazidime [28,29] but their hydrolyzing activity have increased to involve CAZ as a result of point mutations around the active site of some enzymes belonging to the CTX-M-1 and CTX-M-9 groups [30].

High proportion (42.1%) of non-pregnant women's isolates had two types of ESBL genotypes versus only 4.3% of pregnant women's isolates. It seems likely that both genital and intestinal tracts of pregnant and non-pregnant women represent different colonization environments for *E. coli* in terms of types of antibiotics that can be used for treatment of each. During pregnancy there is a limitation of types of antibiotics that should be used in order not to affect the fetus. Therefore, there is differences in selective pressure that selects for emergence of resistance [31].

The risk of this widespread of ESBL-producing *E. coli* strains resides not only in rendering oxyimino-cephalosporins ineffective, but also these organisms pose a therapeutic challenge, since they are frequently resistant to other kinds of antimicrobial drugs, including aminoglycosides, quinolones, and cotrimoxazole [4,20]. The plasmids bearing the genes encoding ESBLs frequently also carry genes encoding resistance to aminoglycosides and trimethioprim/sulfamethoxazole. There have been increasing reports of plasmid-encoded decrease in susceptibility to quinolones, frequently in association with plasmid-mediated cephalosporin resistance. Even when plasmid-encoded decrease in quinolone susceptibility is not present, there is a strong association between quinolone resistance and ESBL production [19]. In this study, 59.2% (50.0% and 61.1% of pregnant and non-pregnant women's isolates, respectively) of ESBL-producing isolates were multidrug resistant (Table 3), showing resistance to gentamicin and kanamicin (37.5% and 43.7%, respectively), ciprofloxcin and norfloxacin (40.6% and 31.2%, respectively), and trimethoprim-sulfamethoxazole (59.3%). In Iraq, aminoglycosides, quinolones and trimethoprim-sulfamethoxazole are widely and heavily used for treatment of different infections, in addition to different types of β-lactams, more than third-generation cephalosporins. This high dependence on these drugs may be responsible for the emergence of ESBL-producing strains rather than the extensive use of third-generation cephalosporins for treatment. Paterson and Bonomo [19] reviewed that the use of a variety of other antibiotic classes (quinolones, trimethoprim-sulfamethoxazole, aminoglycosides, and metronidazole) has been found to be associated with subsequent infections due to ESBL-producing organisms. Conversely, prior use of β-lactam/β-lactamase inhibitor combinations, penicillins, or carbapenems seems not to be associated with frequent infections with ESBL-producing organisms.

Vaginal colonization by ESBL-producing *E. coli* provides a reservoir for future infections both in these women and their contacts. The women themselves are vulnerable to acute and recurrent urinary tract infections with vaginal strains [32] as well as there is the possibility of transferring such strains to their sexual partners [33]. The nosocomial risk of vaginal colonization by such isolates resides in the possibility of creation of an outbreak in the hospital environment especially in the neonatal intensive care unit (NICU) as a result of the transfer of these isolates from the mother to the neonate during delivery. In Switzerland [34], an outbreak of ESBL-producing *E. coli* in a neonatal intermediate care unit was reported. Initial transmission was from a mother to her newborn twins and subsequently by physical contact of health care workers with other patients; an health care worker also was infected. Similar results were reported from Iran, where Feizabadi *et al.*[35] carried out a genetic analysis of ESBL-producing *Klebsiella pneumoniae* nosocomial isolates and found that the clonal spread played a role in the dissemination of ESBL-producing isolates in the NICU, as a result of relatedness of a multi-drug resistant *K. pneumoniae* clone detected in the NICU to a clone isolated from women's surgery, since both of them produced similar pulsed-field gel electrophoresis (PFGE) patterns.

In conclusion, genital tracts of pregnant and non-pregnant women represent different environments for propagation of ESBL-producing *E. coli*. In Iraq, nationwide study is required to make a precise estimation of this widespread of ESBL-producing bacteria.

## Consent

Oral consent was obtained from each patient for collecting specimens and publication of this report.

The reason for just obtaining oral consent without the need for written consent is that collection of high vaginal specimens is part of routine clinical laboratory work for diagnosis of these infections. Specimen collection is confined only to Gynecologists who decided the necessity of collecting such specimens.

## Competing interest

I have no competing interests.
